# Effect of Molybdenum Additives on Corrosion Behavior of (CoCrFeNi)_100−*x*_Mo*_x_* High-Entropy Alloys

**DOI:** 10.3390/e20120908

**Published:** 2018-11-28

**Authors:** Wenrui Wang, Jieqian Wang, Honggang Yi, Wu Qi, Qing Peng

**Affiliations:** 1 School of Mechanical Engineering, University of Science and Technology Beijing, Beijing 100083, China; 2 Nuclear Engineering and Radiological Sciences, University of Michigan, Ann Arbor, MI 48109, USA

**Keywords:** (CoCrFeNi)_100−*x*_Mo*_x_* alloys, high entropy alloy, microstructure, mechanical properties, corrosion behavior

## Abstract

The present work investigates the influence of micro-alloyed Mo on the corrosion behavior of (CoCrFeNi)_100−*x*_Mo*_x_* high-entropy alloys. All of the (CoCrFeNi)_100−*x*_Mo*_x_* alloys exhibit a single face-centered cubic (FCC) solid solution. However, the (CoCrFeNi)_97_Mo_3_ alloy exhibits an ordered sigma (*σ*) phase enriched in Cr and Mo. With the increase of *x* (the Mo content) from 1 to 3, the hardness of the (CoCrFeNi)_100−*x*_Mo*_x_* alloys increases from 124.8 to 133.6 Vickers hardness (HV), and the compressive yield strength increases from 113.6 MPa to 141.1 MPa, without fracture under about a 60% compressive strain. The potentiodynamic polarization curve in a 3.5% NaCl solution indicates that the addition of Mo has a beneficial effect on the corrosion resistance to some certain extent, opposed to the *σ* phase. Furthermore, the alloys tend to form a passivation film in the 0.5 M H_2_SO_4_ solution in order to inhibit the progress of the corrosion reaction as the Mo content increases.

## 1. Introduction

Traditional alloys only have one major element as a matrix [1]. With the increase of the amount of alloying elements and the concentration of minor elements, the alloy forms a fragile intermetallic phase, which not only increases the difficulty of the microstructure, but also may result in a reduction in the mechanical performance [2,3,4]. To overcome these difficulties, high entropy alloys (HEAs) are invented with extensive research interests; HEAs usually compose of five or more kinds of major elements, with the concentration of each principal element being between 5 and 35 at % [5,6,7]. HEAs tend to generate a face-centered cubic (FCC), body-centered cubic (BCC), or hexagonal closed-packed (HCP) multicomponent solid solution phase [8,9]. Some HEAs have been confirmed to achieve a series of excellent properties, such as high strength, high hardness, and glorious corrosion resistance [10,11,12].

The CoCrFeNi alloy has received extensive attention for its outstanding corrosion resistance, ductility, and structure stability [13,14]. However, because of the poor mechanical strength, the application of the CoCrFeNi alloy in engineering has been limited. It has been shown that an ordered σ strengthening phase can be formed by a certain amount of Mo additives, resulting in precipitation strengthening [15,16,17]. However, the excessive addition of Mo causes a large amount of the coarse *σ* strengthening phase, which may lead to a rapid increase of the alloy brittleness. Furthermore, because of the low electronic potential of Mo, the excessive content of Mo may reduce the corrosion resistance [18,19,20]. Referring to the chemical composition of austenitic stainless steel, the content of Mo in stainless steel is generally less than 3 wt %. Therefore, we also supply a small amount of Mo in addition to the CoCrFeNi alloy, in order to study the corrosion resistance of the (CoCrFeNi)_100−*x*_Mo*_x_* alloy for the development of an HEA system, with a good performance of both strength and corrosion resistance.

In this paper, the as-cast (CoCrFeNi)_100−*x*_Mo*_x_* (*x* =1, 2, and 3 wt %) HEAs have been prepared mainly by vacuum arc melting. The excellent corrosion behavior was investigated by the electrochemical experiments.

## 2. Materials and Methods

### 2.1. Samples Fabrication

Elements Co, Cr, Fe, Ni, and Mo with purities of over 99.9 wt % were prepared as raw materials previously. The as-cast (CoCrFeNi)_100−*x*_Mo*_x_* (*x* = 1, 2, and 3, represented by Mo_1_, Mo_2_, and Mo_3_, respectively) were prepared by vacuum arc melting and were fabricated under the WK-∏ vacuum arc melting furnace at least five times in the crucible, so as to ensure the chemical homogeneity. The size of the ingot was approximately Φ 35 × 10 mm, and the ingot was annealed for 3 h under 500 °C and was cooled in the air so as to release the residual stress caused by rapid cooling during casting.

### 2.2. Microstructure of the (CoCrFeNi)_100−x_Mo_x_ Alloys

The crystalline phases of the (CoCrFeNi)_100−*x*_Mo*_x_* alloys were identified by X-ray diffraction (XRD), using an Ultima IV X-ray diffractometer with Cu K*_α_* radiation. The X-ray diffractometer has an operating voltage of 30 kV and an operating current of 20 mA with the diffraction angle (2*θ*) from 20 to 90°, at a scanning rate of 4°/min.

Etching the (CoCrFeNi)_100−*x*_Mo*_x_* alloys with aqua regia (HNO_3_: HCl = 1:3, volume fraction) and using FE-SEM JEOL JEM-7600F scanning-electron microscopy (SEM) (JEOL Ltd., Tokyo, Japan) equipped with an energy dispersive spectrometer (EDS) allowed for an analysis of the microstructure and composition.

### 2.3. Mechanical Properties

The microhardness was measured using a Wolpert-401MVD Vickers hardness tester (WOLPERT Co., Norwood, MA, USA) with loads of 500 g and a duration of 10 s. The measurements were performed at 10 different locations on each sample, and the average value of the 10 measurements was calculated. Compressive tests were carried out on the Φ 3 × 6 mm samples, using the universal testing machine (CMT 4305) (MTS Co., Eden Prairie, MN, USA) with a strain rate of 10^−3^ s^−1^.

### 2.4. Electrochemical Corrosion Test

The electrochemical experiments were performed on (CoCrFeNi)_100−*x*_Mo*_x_* alloys, using the Potentiostat Workstation Versa STAT MC (PARSTAT 4000, AMETEK Co., Princeton, NJ, USA). A three-electrode electrochemical cell using a saturated calomel electrode (SCE) as a reference electrode, a platinum plate as an auxiliary electrode, and a sample as a working electrode were tested. The electrochemical experiments of the (CoCrFeNi)_100−*x*_Mo*_x_* alloys were conducted in a 3.5% NaCl and 0.5 M H_2_SO_4_ solution at room temperature, respectively. The potentiodynamic polarization measurements were taken at a scan rate of 1 mV/s from a potential scanning range of −0.5 V to 1.1 V.

### 2.5. Corroded Microstructure

The electrochemically tested alloys were cleaned using an ultrasonic cleaner, and then dried in nitrogen. SEM and EDS were used to study the morphology of the corrosion surface of the high-entropy alloys.

## 3. Results and Discussion

### 3.1. Microstructure of the (CoCrFeNi)_100−x_Mo_x_ Alloys

**Figure 1:**
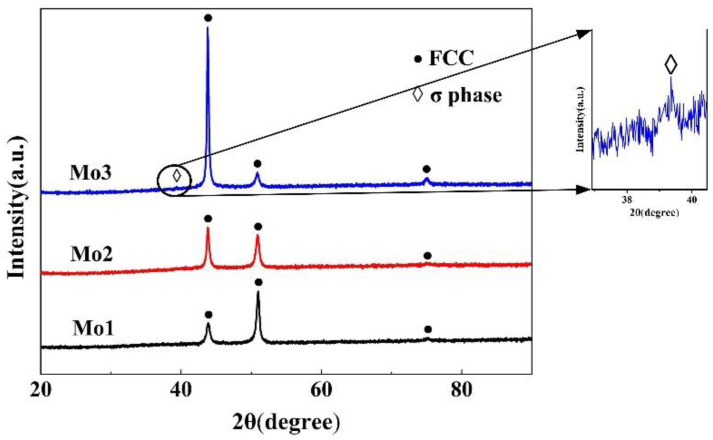
XRD (X-ray diffraction) patterns of the (CoCrFeNi)_100−*x*_Mo*_x_* (*x* = 1, 2, 3) high entropy alloys.

Figure 1 represents the XRD pattern of the (CoCrFeNi)_100−*x*_Mo*_x_* (*x* = 1, 2, 3) alloys. All of the samples of alloys have a single FCC solid solution structure, which was confirmed by the predecessors [21]. As the Mo content increases, the peak intensity changes, but the FCC phase is kept. As the Mo content becomes 3 wt % (Mo_3_ structure), the small peak on the left of the matrix FCC phase in the XRD patterns is identified as Cr and Mo rich *σ* phase, which agrees with the previous remarks [22].

**Figure 2:**
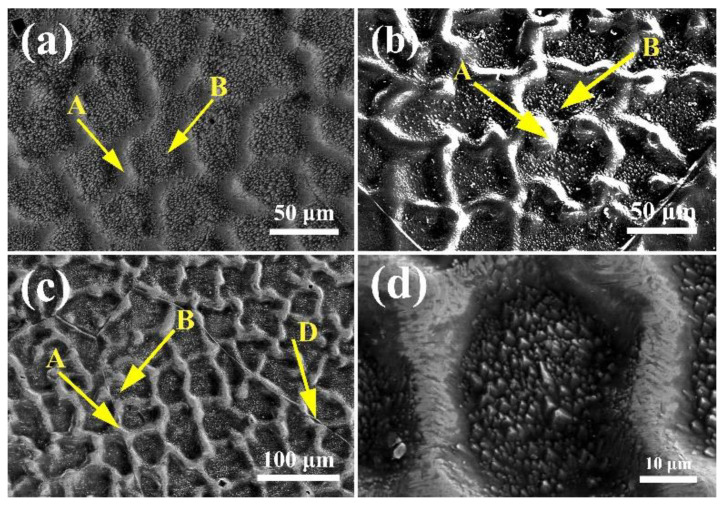
SEM (scanning-electron microscopy) images of the (CoCrFeNi)_100−*x*_Mo*_x_* high-entropy alloys: (**a**) *x* = 1; (**b**) *x* = 2; (**c**) *x* = 3; (**d**) 10 × magnification.

The SEM images of the (CoCrFeNi)_100−*x*_Mo*_x_* (*x* = 1, 2, and 3) alloys presented in Figure 2 shows that the alloys are composed of typical dendritic structures. Region A is interdendrites and region B is dendrites. Figure 2c presents the SEM image of the Mo_3_ alloy, and region D is the grain boundary. Figure 2d is a part of the SEM of the Mo_3_ alloy. The EDS results of the (CoCrFeNi)_100−*x*_Mo*_x_* alloys are shown in Table 1. According to the EDS, the dendrite is the Co and Fe rich phase, and the interdendrite is the Cr and Mo rich phase. Combined with the XRD and EDS results, the Mo_3_ alloy exhibits Cr and Mo rich σ phase in the interdendrite. When the content of Mo is 1 and 2 wt %, there is no formation of a precipitate phase in the alloy because of the high entropy effect. However, HEAs undergo spinodal decomposition inside the crystal grains during cooling, leading to the formation of microstructures with the same structure but different compositions.

### 3.2. Mechanical Properties

**Figure 3:**
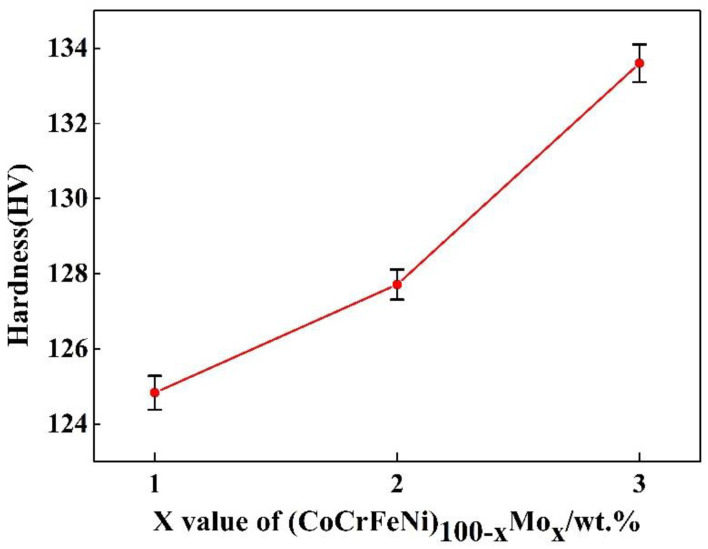
Vickers hardness of the (CoCrFeNi)_100−*x*_Mo*_x_* high-entropy alloys as a function of the Mo content.

Figure 3 shows the Vickers hardness (HV) of the (CoCrFeNi)_100−*x*_Mo*_x_* alloys as a function of the Mo content. The alloy hardness increases from 124.83 to 133.60 HV, with the Mo content increasing from 1 to 3 wt %. When the content of the Mo element is 3 wt %, the presence of the σ phase results in a remarkable increase in the hardness.

**Figure 4:**
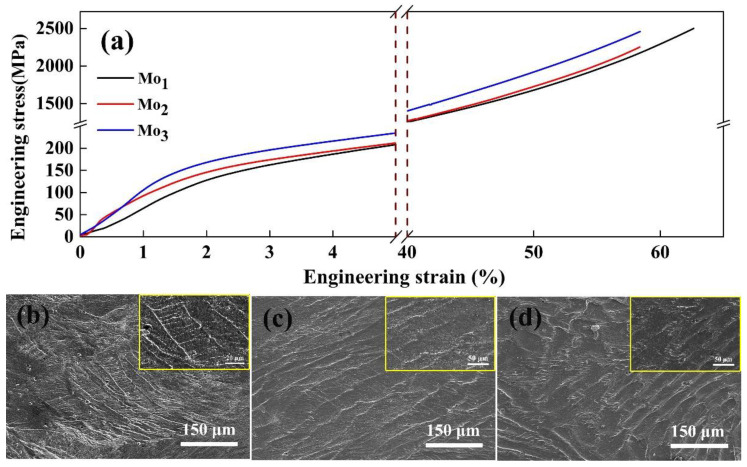
(**a**) Compressive stress–strain curves and the inner longitudinal-section SEM images of the (CoCrFeNi)_100−*x*_Mo*_x_* alloys after compression deformation: (**b**) *x* = 1; (**c**) *x* = 2; (**d**) *x* = 3.

Besides the hardness, we have examined the stress–strain relationships. The compressive stress–strain curves and the inner longitudinal-section SEM images of the (CoCrFeNi)_100−*x*_Mo*_x_* (*x* = 1, 2, and 3) alloys are shown in Figure 4. After yielding, the strength of the alloys increases continuously. All of the three samples do not break under about a 60% compressive strain, indicating that the alloys possess a good ductility, flexibility, and fracture strain. As shown in Figure 4b–d, the deformation of the Mo_3_ subgrain boundaries is more prominent in the angle of 45°, probably due to the resolved shear stress.

Table 2 lists the mechanical properties of the (CoCrFeNi)_100−*x*_Mo*_x_* alloys. The Mo_1_ and Mo_2_ alloys exhibit a similar behavior. The yield stress of the Mo_3_ alloy increases significantly because of the second-phase hardening by the σ phase, as reported in the literature [23,24].

### 3.3. Environmental Effect on Corrosion Behavior

#### 3.3.1. Corrosion Behavior in Chloride-Containing Solutions

**Figure 5:**
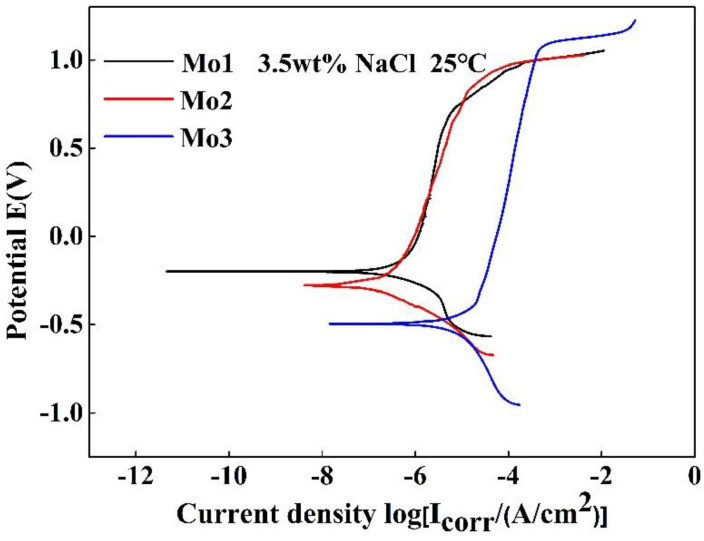
Polarization curves of the (CoCrFeNi)_100−*x*_Mo*_x_* (*x* = 1, 2, and 3) alloys in a 3.5% NaCl solution.

Figure 5 lists the polarization curve of the (CoCrFeNi)_100−*x*_Mo*_x_* (*x* = 1, 2, and 3) alloys in a 3.5% NaCl solution. The corrosion potential of the alloys gradually shifts to more positive potentials with a decreasing Mo content.

Table 3 presents the electrochemical parameters of the (CoCrFeNi)_100−*x*_Mo*_x_* (*x* = 1, 2, and 3) alloys in a 3.5% NaCl solution. The corrosion current densities (*i_corr_*) of the Mo_1_, Mo_2_, and Mo_3_ alloys were 0.4, 0.24, and 6.6 μA/cm^2^, and the corrosion potentials (*E_corr_*) were −199, −277, and −493 mV, respectively. The breakdown potential (*E_b_*) gradually shifts to more positive potentials. *E_pit_* is a primary passivation potential. ∆*E* is the passive region width, defined as the difference between the *E_b_* and *E_pit_*. The *i_corr_* value of the Mo_3_ alloy is an order of magnitude higher than the other two. The corrosion resistance of Mo_3_ was dropped. Therefore, the presence of the Cr and Mo rich *σ* phase in the Mo_3_ alloy leads to the diminution of the corrosion resistance.

**Figure 6:**
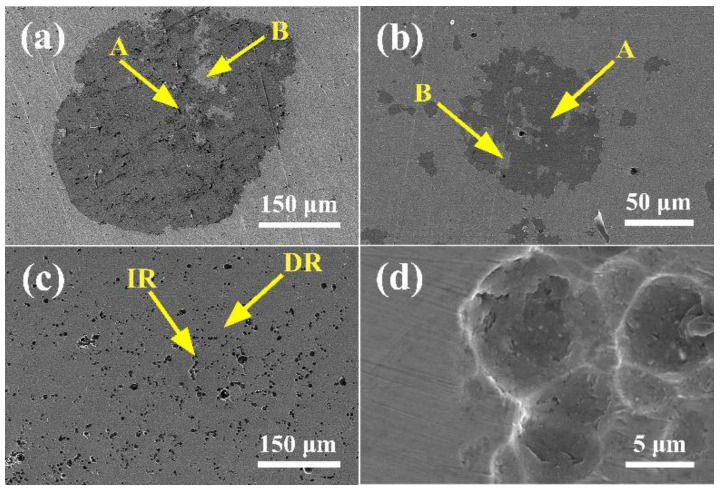
SEM images of (CoCrFeNi)_100−*x*_Mo*_x_* after potentiodynamic polarization in a 3.5% NaCl solution: (**a**) *x* = 1; (**b**) *x* = 2; (**c**) *x* = 3; (**d**) the interdendrite morphology of the Mo_3_ alloy.

Figure 6 presents the microstructure of the (CoCrFeNi)_100−*x*_Mo*_x_* alloys after potentiodynamic polarization in a 3.5% NaCl solution. DR is dendrite and IR is interdendrite. According to the SEM images, the majority of types of corrosion were mainly the pitting corrosion. The Mo_3_ alloy is more susceptible to pitting corrosion, which is consistent with the polarization curve results. Region A is a Cr-rich phase and region B is a Mo-rich phase. There is an element of segregation that causes the corrosion to occur. The interdendritic phase of Mo_3_ is the Cr- and Mo-rich phase, and the dendrite is a Cr- and Mo-depleted phase. Therefore, galvanic corrosion occurred at the junctions of dendrites. The XRD and SEM result show that the Mo and Cr rich *σ* phase appeared inside the interdendrite, the corrosion occurred at the interfaces around *σ* phase, as shown in Figure 6c,d. The results of the EDS of the alloy after corrosion are summarized in Table 4.

#### 3.3.2. Corrosion Behavior in Acid Solutions

**Figure 7:**
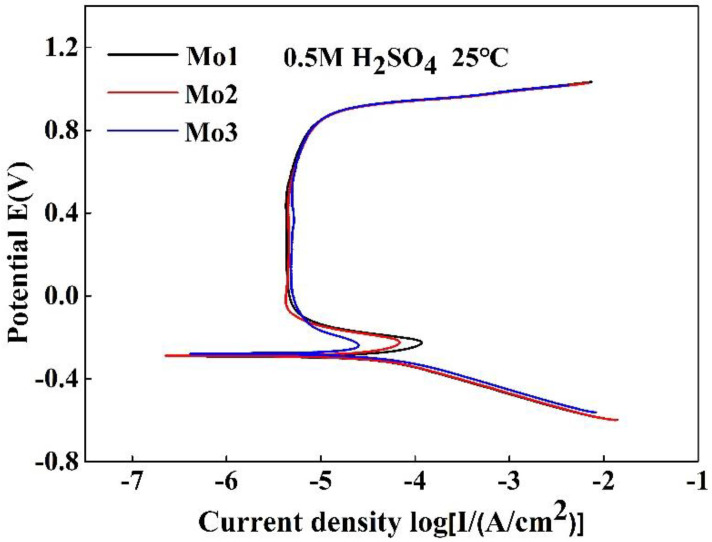
Polarization curves of the (CoCrFeNi)_100−*x*_Mo*_x_* (*x* = 1, 2, and 3) alloys in a 0.5 M H_2_SO_4_ solution

Figure 7 shows the polarization curve of the (CoCrFeNi)_100−*x*_Mo*_x_* (*x* = 1, 2, and 3) alloys in 0.5 M H_2_SO_4_. Table 5 presents the electrochemical parameters of the (CoCrFeNi)_100−*x*_Mo*_x_* (*x* = 1, 2, and 3) alloys in 0.5 M H_2_SO_4_. The *i_pp_* is the lunt current density. It can be seen from the electrochemical parameters that the *i_corr_* was 34.1, 28.0, and 15.4 μA/cm^2^, respectively. The *E_corr_* shifted to more positive potentials, and the *i_corr_* value dropped as the Mo content increased. This suggests that the alloys tend to form a passivation film to inhibit the progress of the corrosion reaction.

**Figure 8:**
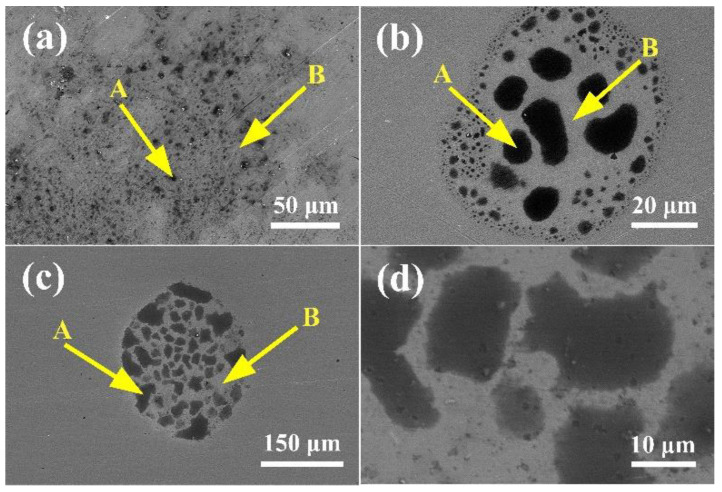
SEM images of (CoCrFeNi)_100−*x*_Mo*_x_* after potentiodynamic polarization in a 0.5 M H_2_SO_4_ solution: (**a**) *x* = 1; (**b**) *x* = 2; (**c**) *x* = 3; (**d**) partial view of the Mo_3_ alloy.

Figure 8 shows the SEM microstructure of the (CoCrFeNi)_100−*x*_Mo*_x_* alloys after potentiodynamic polarization in a 0.5 M H_2_SO_4_ solution, combined with EDS, because the low potential of Mo is enriched and the Cr_2_O_3_ is insufficient in region A. Consequently, region A is more susceptible to corrosion in the H_2_SO_4_ solution. When the content of Mo is 3 wt %, as Figure 8c indicates, the results of the EDS analysis show that the concentration of element Cr in region A is reduced, and the content of Mo in region B is significantly higher than that in region A, the effect of Mo was to form Mo (VI) oxyhydroxide or molybdate (MoO_4_^2−^), decreasing the rate of dissolution in active zones. Hence, the corrosion of the Mo_3_ alloy is concentrated in region A.

**Figure 9:**
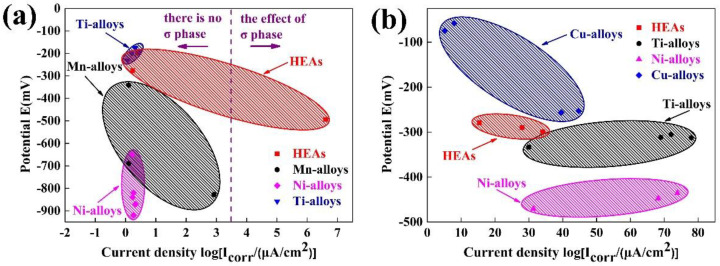
Comparison of the *i_corr_* and *E_corr_* between high entropy alloys (HEAs) and conventional alloys: (**a**) in a 0.5 M NaCl solution; (**b**) in a 0.5 M H_2_SO_4_ solution.

Figure 9a shows the comparison of the corrosion behavior between the HEAs and the conventional corrosion resistant alloys in a 3.5% NaCl solution, compared with those of the conventional corrosion resistant alloys [25,26]. The HEAs are located in the upper part of Figure 9a, the *E_corr_* of the HEAs are more positive than those of the Mn alloys, Ni alloys, and some of the Ti alloys. On the other hand, the *i_corr_* of the HEAs are much lower than some of Mn alloys and are comparable with the Ti alloys, which indicates that the corrosion resistance of the HEAs is comparable or even better than those of the conventional alloys. However, the partial *i_corr_* of the HEAs is higher than that of the total, because the presence of the σ phase is catastrophic for HEAs. Figure 9b presents the comparison of the corrosion behavior between the HEAs and the conventional corrosion resistant alloys in the 0.5 M H_2_SO_4_ solution. Compared with the conventional alloys [27,28], the *E_corr_* of the HEAs are much more positive than those of the Ti alloys and Ni alloys. The *i_cor_*_r_ of the HEAs are much lower than the Ti alloys, Ni alloys, and some of the Cu alloys. As a general trend, the corrosion resistance of the HEAs in the 0.5 M H_2_SO_4_ solution is better than those of the conventional alloys.

## 4. Conclusions

The (CoCrFeNi)_100−*x*_Mo*_x_* (*x* = 1, 2, and 3) alloys have been synthesized. Their microstructures, mechanical properties, and corrosion behaviors have been experimentally investigated. The microstructures of the (CoCrFeNi)_100−*x*_Mo*_x_* (*x* = 1, 2, and 3) alloys belong to a single FCC structure. The increase in Mo promotes the formation of the Cr- and Mo-rich σ phase. The hardness and compressive yield strength increase obviously with an increase of the Mo content from 1 to 3 wt %. Regarding the potentiodynamic polarization curves of the (CoCrFeNi)_100−*x*_Mo*_x_* alloys in a 3.5% NaCl solution, the curves of the Mo_1_ and Mo_2_ alloys indicated that the increase of the Mo content increases the corrosion resistance of the chloride environment to some extent. However, the Cr and Mo rich *σ* phase is present at the grain boundary of the Mo_3_ alloy, resulting in a decreasing in corrosion resistance in the 3.5% NaCl solution. Furthermore, the potentiodynamic polarization curves of the (CoCrFeNi)_100−*x*_Mo*_x_* alloys in the 0.5 M H_2_SO_4_ solution yielded an extensive passive region, and as the content of the Mo increased, the corrosion current density gradually decreased. Therefore, in an acidic solution, the addition of Mo has a positive effect on the corrosion resistance of the (CoCrFeNi)_100−*x*_Mo*_x_* alloys.

## Figures and Tables

**Table 1:** Element concentration determined using the energy dispersive spectrometer (EDS) of the three samples of the (CoCrFeNi)_100−*x*_Mo*_x_* alloys (at %).

Element		Cr	Fe	Co	Ni	Mo
Mo_1_	Dendrite	25.25	23.54	26.07	24.53	0.61
	interdendrite	27.69	22.31	24.03	24.94	1.04
Mo_2_	Dendrite	26.02	25.17	24.55	23.56	0.69
	interdendrite	27.20	23.20	23.33	23.87	2.40
Mo_3_	Dendrite	24.99	23.47	25.28	24.96	1.31
	interdendrite	26.82	22.54	24.81	24.35	1.50

**Table 2:** Mechanical properties (yield stress, compressive strength, and fracture strain) of the (CoCrFeNi)_100−*x*_Mo*_x_* alloys for *x* = 1, 2, and 3 as Mo_1_, Mo_2_, and Mo_3_, respectively.

Alloy	Yield Stress *σ_y_* (MPa)	Compressive Strength σ_max_ (MPa)	Fracture Strain *ε_p_* (%)
Mo_1_	113.6	Not fractured	>60
Mo_2_	119.7	Not fractured	>60
Mo_3_	141.1	Not fractured	>60

**Table 3:** Electrochemical parameters of the (CoCrFeNi)_100−*x*_Mo*_x_* alloys in a 3.5% NaCl solution.

Alloy	*E_corr_* (mV)	*I_corr_* (μA/cm^2^)	*E_b_* (mV)	*E_pit_* (mV)	∆*E* (mV)
Mo_1_	−199	0.402	992	−5	997
Mo_2_	−277	0.235	968	−108	1076
Mo_3_	−493	6.610	1053	−358	1411

**Table 4:** EDS results for the Mo_3_ alloy after the potentiodynamic polarization in a 3.5% NaCl solution.

Element	Region	Cr (%)	Fe (%)	Co (%)	Ni (%)	Mo (%)
Mo_1_	A	26.17	24.56	23.32	25.01	0.93
	B	30.79	23.79	22.87	22.51	0.04
Mo_2_	A	26.44	23.72	24.32	23.83	1.70
	B	35.81	23.79	19.78	20.11	0.52
Mo_3_	DR	24.96	25.21	24.72	24.88	0.23
	IR	29.06	23.21	22.43	22.76	2.54

**Table 5:** Electrochemical parameters of the (CoCrFeNi)_100−*x*_Mo*_x_* alloys in a 0.5 M H_2_SO_4_ solution.

Alloy	*E_corr_* (mV)	*I_corr_* (μA/cm^2^)	*E_pp_* (mV)	*i_pp_* (μA/cm^2^)	∆*E* (mV)
Mo_1_	−294	34.1	−225	117	655
Mo_1_	−290	28.0	−225	68	681
Mo_1_	−279	15.4	−239	25	751
